# Detection and Identification of Defects in 3D-Printed Dielectric Structures via Thermographic Inspection and Deep Neural Networks

**DOI:** 10.3390/ma14154168

**Published:** 2021-07-27

**Authors:** Barbara Szymanik, Grzegorz Psuj, Maryam Hashemi, Przemyslaw Lopato

**Affiliations:** 1 Center for Electromagnetic Fields Engineering and High-Frequency Techniques, Faculty of Electrical Engineering, West Pomeranian University of Technology, 70-310 Szczecin, Poland; Grzegorz.Psuj@zut.edu.pl (G.P.); Przemyslaw.Lopato@zut.edu.pl (P.L.); 2 Department of Electrical Engineering, Iran University of Science and Technology, Teheran 13114-16846, Iran; maryam_hashemi@elec.iust.ac.ir

**Keywords:** active thermography, deep learning, convolutional neural networks, 3D-Printed structure quality

## Abstract

In this paper, we propose a new method based on active infrared thermography (IRT) applied to assess the state of 3D-printed structures. The technique utilized here—active IRT—assumes the use of an external energy source to heat the tested material and to create a temperature difference between undamaged and defective areas, and this temperature difference is possible to observe with a thermal imaging camera. In the case of materials with a low value of thermal conductivity, such as the acrylonitrile butadiene styrene (ABS) plastic printout tested in the presented work, the obtained temperature differences are hardly measurable. Hence, the proposed novel IRT method is complemented by a dedicated algorithm for signal analysis and a multi-label classifier based on a deep convolutional neural network (DCNN). For the initial testing of the presented methodology, a 3D printout made in the shape of a cuboid was prepared. One type of defect was tested—surface breaking holes of various depths and diameters that were produced artificially by inclusion in the printout. As a result of examining the sample via the IRT method, a sequence of thermograms was obtained, which enabled the examination of the temporal representation of temperature variation over the examined region of the material. First, the obtained signals were analysed using a new algorithm to enhance the contrast between the background and the defect areas in the 3D print. In the second step, the DCNN was utilised to identify the chosen defect parameters. The experimental results show the high effectiveness of the proposed hybrid signal analysis method to visualise the inner structure of the sample and to determine the defect and size, including the depth and diameter.

## 1. Introduction

Three-dimensional (3D) printing, also known as additive manufacturing (AM) is defined as the fabrication of a 3D object from a non-physical concept, usually layer by layer. This approach was first described in Hull [1]. Currently, 3D printing has become widely embraced for different industrial applications. In AM technology, there is a trade-off between the high cost of the equipment and the quality, i.e., complex-shaped and high-quality products can be fabricated, although they are usually costly and require relatively longer production times [2]. There is a critical need to develop printable polymer composites with high performances owing to the intrinsically limited mechanical properties and functionalities of printed pure polymer parts.

Despite these problems, 3D printing has been broadly used for product design in several industries, including, but not limited to, the medical (for building blood vessels or low-cost prosthetic parts), architectural, and automotive industries. This vast utilisation is consequent to the numerous advantages of 3D printing in the fabrication of composites, i.e., high precision, cost-effectiveness, and customised geometry manufacturing possibilities [3,4].

In the last decade, there have been considerable efforts to evaluate the quality of materials produced using AM, and various techniques have been introduced. Non-destructive testing (NDT) methods are one of the most important approaches. Notably, NDT methods are employed to identify and characterise the surface and internal damage of a material without any physical interactions, such as cutting or altering. In addition to these advantages, NDT methods are designed to be a cost-effective means of system quality control [5,6]. Generally, NDT methods can be divided into contact and non-contact techniques.

Both approaches are utilised in specific applications for testing and evaluating a variety of materials, including 3D prints [7,8,9,10,11,12]. The choice of the NDT method applied for AM purposes depends on the type of filament used for printing, the inspection stage, and its scope. Process control in AM is possible at all its stages—preparatory (examination of the quality of the feedstock material), print stage (control of the printing process), and the final evaluation of the finished product. Herein, we focus on quality control in the final stage of 3D printing production.

Non-destructive analyses of finished AM products entail dimensional accuracy and surface finish assessment, internal structure evaluation, and defect detection. Vision [13], microscopy [14,15], and laser profilometry [16] are among the methods most frequently used to assess the surface quality of printed products. The examination of the internal structure of 3D prints, including the detection of subsurface defects, is conducted mainly with the use of computed tomography [17,18,19,20] and ultrasounds [21,22,23]. Active infrared thermography (IRT), as proposed herein, is relatively rarely used in AM; however, the initial studies are promising [24,25].

Recently, the rapid development of IRT techniques has been observed. These methods are relatively cheap and fast, allowing the examination of bulky batches of a given product or large areas of the structure under the test, requiring neither any special preparation of personnel nor the use of exceptional security sources [26]. Thermography enables the observation of temperature distribution at the examined material surface using sensitive equipment, such as a thermovision camera [27].

Notably, with this technique, the thermal image sequence can be recorded, thereby, allowing the analysis of the temperature variation in the time domain [28]. Therefore, the images and time-dependent signals recorded for each measuring point (image pixel) may be used for further study. Furthermore, IRT techniques can be classified as passive and active methods [29,30].

Here, the active approach is considered. In the active mode, the energy is delivered to the examined system (in contrast to passive methods, which do not assume the use of external energy sources), which has previously been in a thermal equilibrium state. The needed temperature variance can be induced in the examined structure using various devices, including conventional heaters (halogen or flash lamps, IR heaters), microwaves generators, hot air, etc. [31]. As a consequence, the internal defects cause changes in the heat transfer within the material, resulting in measurable differences in temperature between sound and defected areas [27].

The key element influencing the effectiveness of the method is the need to create such a temperature contrast in the tested element that will enable the registration of the material anomaly. Popular filaments used in AM can be classified as good thermal insulators. In the case of materials with a low value of thermal conductivity, such as the acrylonitrile butadiene styrene (ABS) plastic printout tested in the presented work, the heat conduction process is relatively slow, and the observed temperature differences between the healthy areas and the defected ones are hardly measurable, especially in the case of damages, such as very narrow defects or micro cracks [32].

Moreover the quality of signals obtained for these materials is generally very sensitive to heating inhomogeneities, local changes in material density, and proximity to the material edges. Therefore, it is extremely important to define the limits of defect sizes that can be detected and, further, to introduce algorithms for processing measurement data, enable to extract even extremely fine anomalies observed in the temperature distribution at the sample surface. To process the thermographic data obtained during the material quality assessment, numerous techniques are applicable.

Various algorithms have been developed for different experimental techniques; therefore, their effectiveness critically depends on the excitation method used in active thermography. The most frequently utilised image and signal processing procedures can be generally divided into those that require a reference point or area and non-reference ones. Among the reference-based contrast methods, which require the use of the temperature in a sound area, the absolute, running, normal, and standard contrast enhancement can be listed [27,33].

The selection of an undamaged region requires the operator to have a priori knowledge of the tested material, which complicates the automation of the process. Undamaged area selection techniques are also sensitive to heating heterogeneity and other disturbances that often appear in the experimental IRT. Due to these problems, non-reference methods have been developed, which do not require knowledge of the material and indication of the undamaged area but, instead, implement a certain (automatic) procedure that enables reference generation based on the given data set.

Among these methods, we can distinguish non-reference techniques for improving the image contrast based on the analytical solution of the diffusion equation, such as differentiated absolute contrast [34,35], and those based on its numerical solution, i.e., 3D finite difference thermal contrast [36]. In addition, there are techniques based on the statistical analysis of the tested signal, e.g., principal component thermography [28,37,38], and those based on advanced approximation methods: thermographic signal reconstruction (TSR) [39,40] and the gapped smoothing algorithm [41].

Most of the aforementioned methods have been developed for pulse thermography (whereby energy is delivered to the system in the form of an ultra-short pulse, e.g., by means of a flash lamp). Evidently, they are also rather effective in other experimental regimes, but there is no doubt that there is a need to develop new, effective methods of signal analysis that can be used for materials, especially those that are difficult to study, whereby the heat transfer process is relatively slow and limited. The sample analysed in this paper, printed using ABS filament, is a good example of such a material. Our study is aimed to prove that, even in this case, the IRT can be considered as an effective method.

In addition to the task of enhancing the contrast, which can be used to locate defects in the material and the initial assessment of their size, there is a need for highly accurate decision-making algorithms for quantitative evaluation of the examined materials, including their internal structure reconstruction. Therefore, new research methods dedicated to machine learning techniques, such as K-nearest neighbours (based on finding the distances between a query and all the exemplary data points, selecting the specified number of points K closest to the query, and then votes for the most frequent label), decision trees (graph-based classifier that labels input observations by splitting them at different levels through a threshold mechanism), decision forest (ensemble of decision trees in order to achieve less overfitting capabilities), different colour spaces, and the histogram of oriented gradients (HOG—this technique counts occurrences of gradient orientation in localized portions of an signal/image; used as a feature descriptor) [42,43], have been developed.

Many machine learning methods are based on feature extraction by specialists. Thus, the effectiveness of classification or clustering algorithms largely depends on the quality of the features (of the described phenomenon or characteristics) stored in the database, making the extraction process critical. Following these expectations based on machine learning techniques, the recent rapid development of complex multi-layered artificial neural networks (ANNs) can be observed. In general, ANNs try to imitate the human learning process to independently learn features. Thus, in these methods, there is no need for human-based feature extraction. A branch of ANNs, convolutional neural networks (CNNs), uses the convolution function to predict the class of the input. This approach is unique for image and video analyses.

These networks have proven their merits in different cases of computer vision tasks, such as image classification [44], object detection or recognition [45], anomaly detection [46], and video analysis [47]. Furthermore, CNNs have been applied in thermography and other NDT techniques.

Promising defect detection results and quantitative evaluation based on CNNs were recently reported for a variety of materials and methods of examination, including IRT in carbon fibre reinforced polymer analyses [48,49], ground penetrating radar in asphalt pavement examination [50], X-ray computed tomography for medical image evaluation [51], visual methods for defect size recognition in steel elements [52], as well as rails [53], and multi-sensor magnetic field measurements for steel defect evaluation [54].

Herein, we propose a 3D-printed structure (characterized by a low value of thermal conductivity) evaluation scheme using active thermography with halogen lamp excitation in a long pulse regime, and dedicated data processing algorithms. The aim of the work was to conduct a procedure enabling an assessment of the possibility of effective extraction of information about even small defects while maintaining short inspection times. Therefore, two areas of using the obtained data should be distinguished in the presented work.

The first is related to the image processing procedure using the data acquired during full testing period. It allows the enhancement of the contrast between the defect and sound areas and verification of the location of defects in recorded images. The designed experimental sequences of thermograms were first processed using the developed algorithm based on the curve-fitting procedure applied to pixel-wise time domain temperature distributions. Padé approximants [55] of logarithmic functions were used as a basis of the fitted curve, and a cross-correlation procedure was used to obtain the final contrasted signals. This step was utilised for high-accuracy defect localisation in the whole sample but was not a component of final defect identification procedure. Nevertheless, the correct indication of areas is important for database preparation and final evaluation of performance of the proposed procedure.

The second area covered in this paper concerns the final deep CNN-based procedure allowing efficient detection and identification of defect parameters based on the short-term part of the whole acquisition period. In this step, the CNN undertakes three tasks: defect detection (in terms of automatic distinction between the defect and sound areas), defect diameter, and (separately) depth classification on a single recorded frame. In order to maximize the efficiency, three deep convolutional networks, separate for each task, were proposed, and supervised learning was utilised.

## 2. Experiment

In this study, active thermography with halogen lamp excitation was used to examine the 3D-printed sample with a set of artificial defects. Halogen lamps allow for continuous radiation heating of the sample surface, and thereafter, the heat is distributed within the sample by conduction. The conduction rate is highly correlated with material properties (such as the density, heat capacity, or thermal conductivity) and is strongly affected by the defects present in the material. Depending on the experimental mode—reflection or transmission—the heated or opposite side of the sample is observed using a sensitive thermovision camera. Defects can be located and evaluated by analysing the time-varying temperature distributions recorded during the experiments. Moreover, the flaw type might be exhibited as a hot or cold spot in the observed distribution. The temperature contrast between the defect and the surrounding sound area depends on the flaw properties.

The polymer materials used in AM are thermal insulators. Therefore, they can generally be characterised by the high values of heat capacity and low thermal conductivity. Owing to this phenomenon, these materials should be heated in a long step in a continuous manner to obtain a visible temperature contrast between the defect and non-defect areas. Consequently, the halogen lamp or infrared heater appears to be the most effective source for active thermography in AM, especially when the task is to detect and evaluate the internal defects of the material.

### 2.1. Experimental Setup and Utilised Sample

The utilised sample was prepared with an acrylonitrile butadiene styrene (ABS) polymer (Table 1 presents its thermal properties [56]). As previously mentioned, this material is considered to be a thermal insulator; therefore, its heating is a relatively longer process. The printed sample was produced with 100% infill density (using parallel lines pattern). The object is composed of 80 layers—layers 1 and 80 are 0.35 mm thick, whereas internal layers—0.25 mm. The width of the outer tracks was set at 0.4mm, and the rest—0.56 mm. The printing temperature was 195 degC.

**Figure 1:**
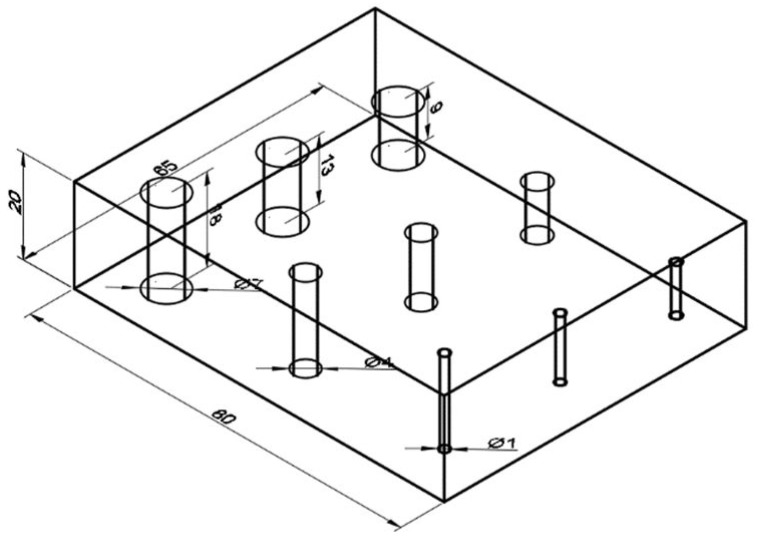
CAD model of the sample.

**Figure 2:**
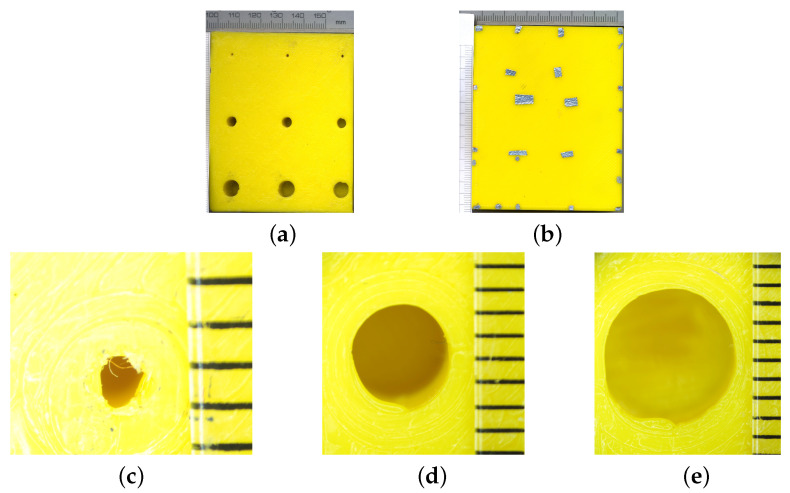
Photos of both sides of the sample (**a**,**b**), close-up of the defects with different diameters (mm): ϕ1 (**c**), ϕ4 (**d**), and ϕ7 (**e**).

Figure 1 and Figure 2 show the geometry of the sample. The sample was printed as an 80 × 65 × 20 mm slab with nine holes of different diameters (ϕ) (1 mm (Figure 2c), 4 mm (Figure 2d), and 7 mm (Figure 2e)) and depths (D) (9, 13, and 18 mm). The holes might be considered as print defects, as well as the planned structure of the manufactured part.

Such low diameter defects located within a thick, thermal insulating material can be considered as extremely hard to detect using IRT. Nevertheless, it will be shown that the IRT can be considered as a suitable method of 3D-print evaluation when it is supported by advanced signal processing and appropriate decision-making algorithms. Our goal was to evaluate the sample in terms of detecting and reconstructing the holes and their geometrical properties.

**Figure 3:**
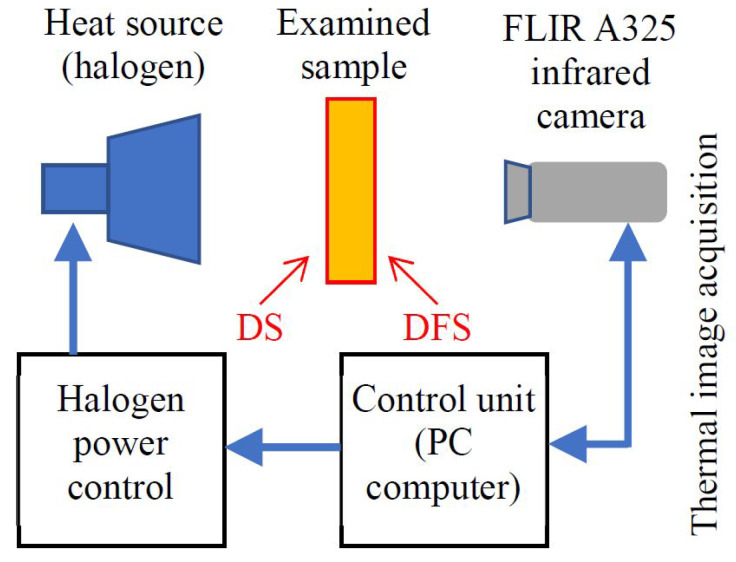
Scheme of the experimental setup; and DS: defect side, DFS: defect-free side.

The experimental setup consisted of a halogen lamp with adjustable power up to 1000 W and a FLIR A325 infrared camera with a close-up lens (Figure 3).

### 2.2. Methodology of the Experiment

In this study, the transmission mode was used (a schematic illustration of the setup is presented in Figure 3). The rear side (defect side—DS (Figure 2a)) of the sample was heated continuously with halogen lamps operating at a power of 500 W, placed approximately 10 cm from the sample, and simultaneously observed using an infrared camera from the opposite side (defect free side—DFS (Figure 2b)).

In this experiment, we decided to use the close-up lens, while significantly decreasing the instantaneous field of view (defined as the angle subtended by the geometrical projection of single detector element to the target surface)—IFOV (100 µm at a distance of 0.07 m), at the cost of decreasing the field of view (determined by the angle of view from the lens out to the scene)—FOV. Consequently, one can record the smaller parts of the sample at high resolution. It is noteworthy that the experiment was repeated for each defect separately. The duration of the heating (in the form of a long pulse) was 60 s.

Subsequently, the heating source was turned off, and the sample was observed for 300 s under natural (convective) cooling. For thermal insulators, the observable changes in temperature are not rapid. Therefore, it was sufficient to record one thermogram per second. As a result, for each defect, 361 thermograms (60 for heating and 301 for cooling) were ready for further processing. To establish the exact region where the defect may be observed, tin foil markers, visible in Figure 2b, were used. These markers were used to divide the whole sample area into small regions that could be observed with a camera equipped with a macro lens.

## 3. Signal Enhancement Algorithm

In our study, the localisation of the defect is critical to correctly assign the description of the defect-free region in the obtained thermograms and to prepare the correct database of the images used as an input of the constructed neural network.

To localise the defect area, many image and data processing techniques can be utilised. As mentioned earlier, the evaluated sample should be considered as a good thermal insulator; as a consequence, the indication of material inhomogeneity is rather weak as the process dynamics are low. Furthermore, a small defect size (ϕ=1), inhomogeneous sample thickness, near-edge defect localisation, and no uniform heating were important factors hindering the detection of defects. Therefore, we, herein, present a new algorithm that improves the visibility of defects based on curve fitting, which may be used in the evaluation of difficult-to-assess materials.

The developed thermal contrast enhancement algorithm is based on analysing the sequence of thermograms pixel-by-pixel to obtain the time–domain temperature curves. It is important to note that, in general, a set of orthogonal logarithmic functions may be used to properly fit the one-dimensional temperature evolution in time (this property is used in the classic TSR method [57]). In most cases, the application of the following polynomial provides excellent agreement with the experimental data:

(1) $$\ln\left(T\left(t\right)\right)=\sum_{n=1}^{N}a_{n}\ln{\left(t\right)}^{n},$$

where T(t) is the time evolution of the temperature, *n* denotes the order of the polynomial, and $a_{n}$ are the polynomial coefficients. The *N* value was adjusted experimentally to fit the data. This approach was originally used for pulsed thermography; however, it was established that it could also be used in other thermography regimes [40]. Therefore, the goal is to use a set of orthogonal functions that fit the temperature curves for the pixels in the sound area with the highest possible rate and have the lowest goodness of fit for the pixels in the defect area.

As the long pulse heating was used, both the heating and cooling phases were used for curve fitting. The idea was to replace the logarithmic functions with their Pad$\overset{´}{e}$ approximants, which were derived by expanding a function as a ratio of two power series of orders *n* and *m*, as well as determining both the numerator and denominator coefficients. Here, the third order logarithmic polynomial was replaced by three Pad$\overset{´}{e}$ approximants of the form [55]:

(2) $$\ln\left(x\right)\approx \frac{2x-2}{x+1}={\mathrm{PA}}_{1}\left(x\right)$$

(3) $$\ln{\left(x\right)}^{2}\approx \frac{{\left(x-1\right)}^{2}}{x}={\mathrm{PA}}_{2}\left(x\right)$$

(4) $$\ln{\left(x\right)}^{3}\approx \frac{2{\left(x-1\right)}^{3}}{3x-1}={\mathrm{PA}}_{3}\left(x\right)$$

**Figure 4:**
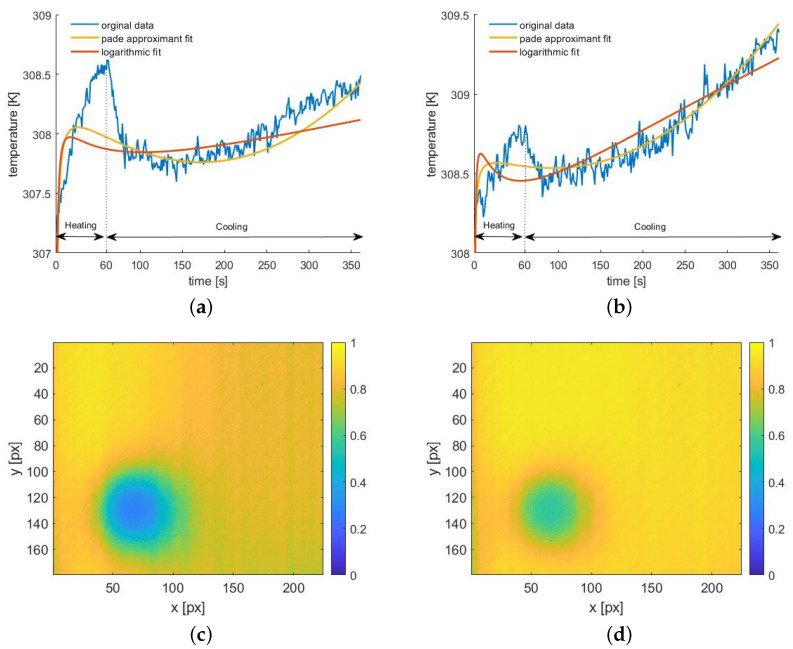
The results of the curve-fitting approach obtained for exemplary defect (having ϕ of 4 mm and D of 18 mm): comparison of the fitting curves obtained using orthogonal logarithmic functions (red line) a Pad$\overset{´}{e}$ approximants (yellow line) for defect (**a**) and sound (**b**) areas; map of the correlation coefficient value obtained for the orthogonal logarithmic functions fit (**c**) and Pad$\overset{´}{e}$ approximants (**d**).

This approach gives a better agreement with the sound area data (correlation coefficient $R^{2}$ equal to 0.91) compared to the third degree logarithmic polynomial ($R^{2}$ = 0.78), as shown in Figure 4. Meanwhile, owing to the use of the Pad$\overset{´}{e}$ approximant, we obtain the desired effect of a low correlation coefficient for data from the defect area ($R^{2}$ = 0.55). The curve mismatch is especially visible within the heating time, i.e., where the impact of the defect on the shape of the function is the most significant.

The correlation coefficient for fitting the third order polynomial is lower in the case of a defect area ($R^{2}$ = 0.27), but the shape of the curve does not reflect the shape of the original function as much as the curve obtained using the Pad$\overset{´}{e}$ approximant (Figure 4a), which results in poorer selectivity. Figure 4b shows a similar comparison of these two approximation curves with the experimental data obtained for the selected pixel from the area without a defect.

Again, we see a better fit of the Pad$\overset{´}{e}$ approximant curve to the experimental data. The maps of the $R^{2}$ coefficient for all the pixels are shown in Figure 4c,d. Despite the lower values of the correlation coefficient in the defect area for the third degree logarithmic polynomial, $R^{2}$ in the sound area is significantly lower than in the case of the Pad$\overset{´}{e}$ approximant. For our purpose, obtaining $R^{2}\approx 1$ in the sound area was the most important goal.

**Figure 5:**
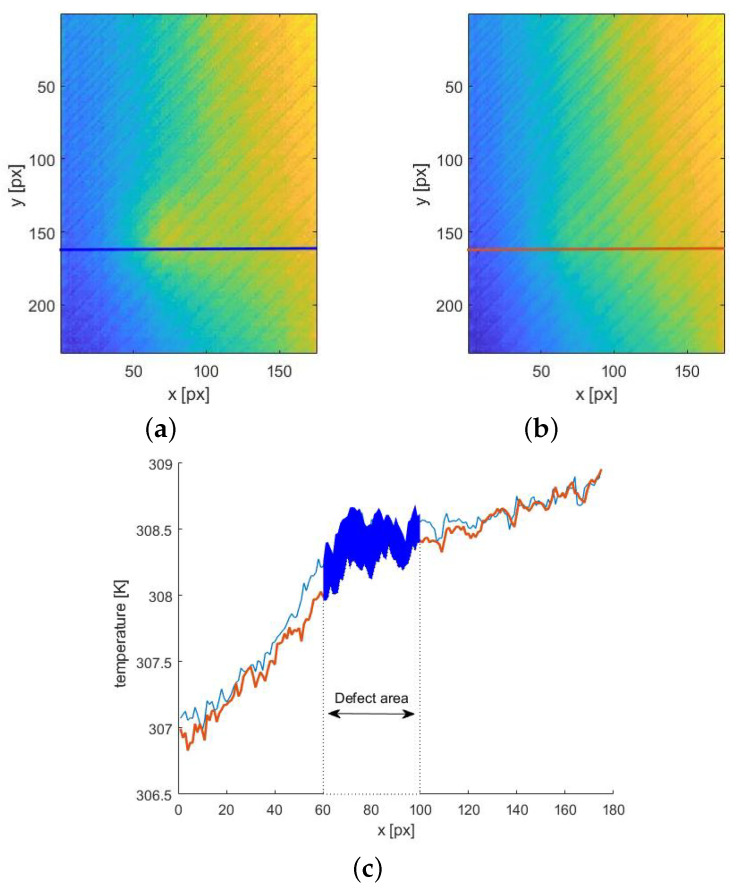
The results of the defect visibility enhancement procedure for the exemplary thermogram registered for defects having ϕ of 4 mm and D of 18 mm for t = 60 s: The original thermogram (**a**) and corresponding 2D distribution of the curve-fitting procedure (**b**); (**c**) presentation of the original and approximated 1D distribution along the lines depicted in (**a**,**b**)—the coloured area marks the difference between the data in the defect zone. The dashed lines depicting the defect area correspond to the real localization of defects in the examined sample.

Using the described approach, by providing the possible best fit for the sound area and possible worst fit for the defect area simultaneously, the background temperature distribution may be retrieved. Therefore, to obtain the visible defect, it should be sufficient to subtract the fitted sequence from the original data. This operation is presented for an exemplary thermogram in Figure 5. It is evident that, in the thermogram reconstructed from the fitted data, the defect signature is not visible—Figure 5b (compared with the original data (Figure 5a)).

The plot along the indicated in Figure 5a,b line, crossing the defect, shows the difference between the original and fitted data in the defect area (Figure 5c. It is important to notice the good compatibility between functions in the sound region. For small and deeper located defects, the obtained differences are small; therefore, their detection is hindered owing to the low signal-to-noise ratio. Thus, the resulting differential sequence was integrated to amplify these small differences. The cumulative integral was used to obtain the sequence of integrals corresponding to the sequence of differential images:

(5) $$A_{I}\left(t_{n}\right)=\int_{t_{0}}^{t_{n}}\left(T\left(t\right)-T_{\mathrm{fit}}\left(t\right)\right)dt$$

where T(t) is the original time–domain function of temperature and $T_{\mathrm{fit}}\left(t\right)$ is the fitted function, while $t_{n}$ denotes the $n_{th}$ time step in the sequence.

The image sequence obtained in the process of fitting the approximated curve is, as mentioned earlier, a reconstruction of the background temperature distribution, wherein non-uniformity is associated with uneven heating, proximity of the edges, and differences in material thickness. Therefore, the reconstructed sequence reproduces the background and excludes the defect. Therefore, its analysis can also provide information on the flaw location. It is natural that, in the curve-fitting process, we obtain the low-pass filtration of the original data. Good results, isolating the defect and reducing background noise, can be obtained by analysing the time derivatives of the approximated signal:

(6) $$A_{D}\left(t\right)=\frac{dT_{\mathrm{fit}}\left(t\right)}{dt}$$

**Figure 6:**
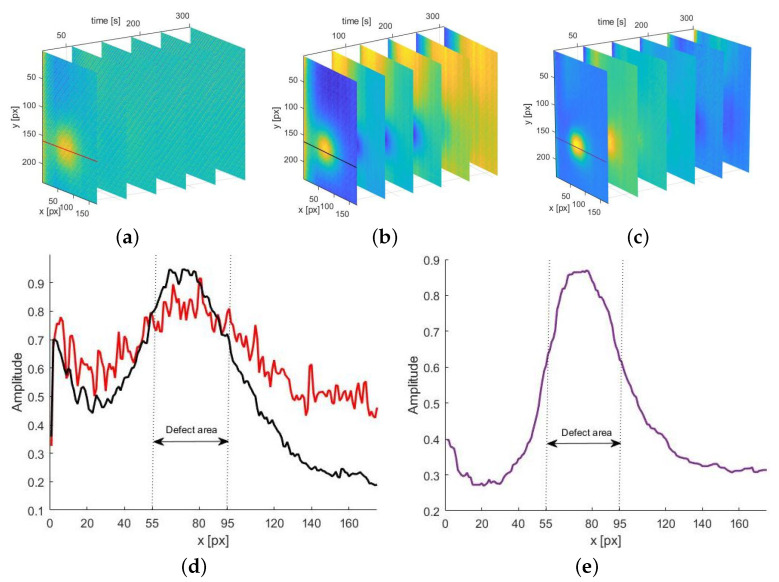
The results of the process of fitting the approximation curves obtained for the chosen defect (ϕ of 4 mm and D of 18 mm): set of the resulting sequences for the defect: (**a**) $A_{I}$ (**b**), $A_{D}$ (**c**) $A_{\mathrm{Res}}$; comparison of distribution of values along the line crossing the defect obtained for corresponding images in a sequences of: (**d**) $A_{I}$ (red line), $A_{D}$ (black line), and (**e**) $A_{\mathrm{Res}}$.

The result of using the procedures described above, i.e., the sequences $A_{I}\left(t_{n}\right)$ and $A_{D}\left(t_{n}\right)$ for exemplary experimental data (i.e., thermograms for defect ϕ 4 D 18), are shown in Figure 6. In both sequences, the defect is visible. However, in the case of the sequence $A_{I}\left(t_{n}\right)$, the signal-to-noise ratio is low, and the flaw is visible to a fairly low degree (Figure 6a).

In the case of the sequence $A_{D}\left(t_{n}\right)$, the background is not completely levelled (Figure 6b). The improvement of the result quality is possible here by applying a cross-correlation procedure. This operation allows us to compare two time signals and to obtain a signal indicating similarities between the input data. First, the sequences $A_{I}\left(t_{n}\right)$ and $A_{D}\left(t_{n}\right)$ were normalised, and then the following operation was applied [58]:

(7) $$A_{\mathrm{Res}}\left(x\right)=\left(A_{I}\times A_{D}\right)\left(x\right)=\sum_{t}A_{I}\left(t\right)A_{D}\left(x+t\right)$$

The resulting sequence is presented in Figure 6c. To demonstrate the effectiveness of this procedure, a comparison of the measured values on the line running through the defect centre for selected images from all sequences is visualised. In Figure 6d, the results for the sequences $A_{I}$ and $A_{D}$ are superimposed, and Figure 6e shows the result of the cross-correlation application. The resulting curve evidently reveals the defect, and the background temperature fluctuations are levelled.

The dashed lines depicting the defect area correspond to the real localization of defects in the examined sample.

**Figure 7:**
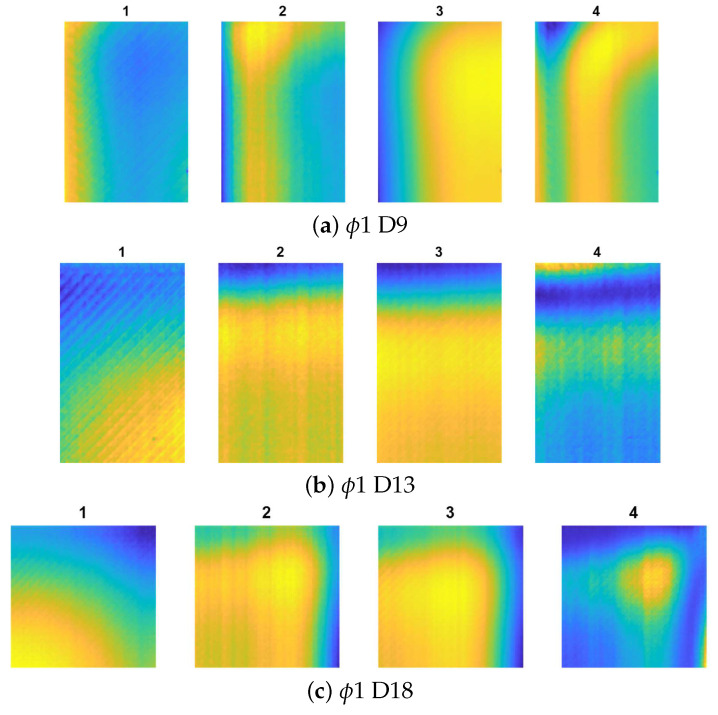
The results of defect visualisation for defects ϕ1 and different D values: 1—raw thermogram (captured after the heating phase t = 60 s), 2—chosen result from sequence $A_{I}$, 3—chosen result from sequence $A_{D}$, and 4—chosen result from sequence $A_{\mathrm{Res}}$.

**Figure 8:**
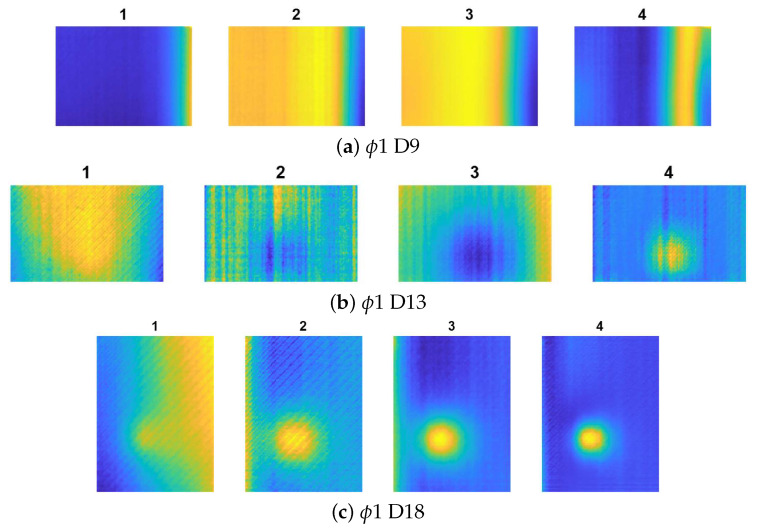
The results of defect visualisation for defects ϕ4 and different D values: 1—raw thermogram (captured after the heating phase t = 60 s), 2—chosen result from sequence $A_{I}$, 3—chosen result from sequence $A_{D}$, and 4—chosen result from sequence $A_{\mathrm{Res}}$.

**Figure 9:**
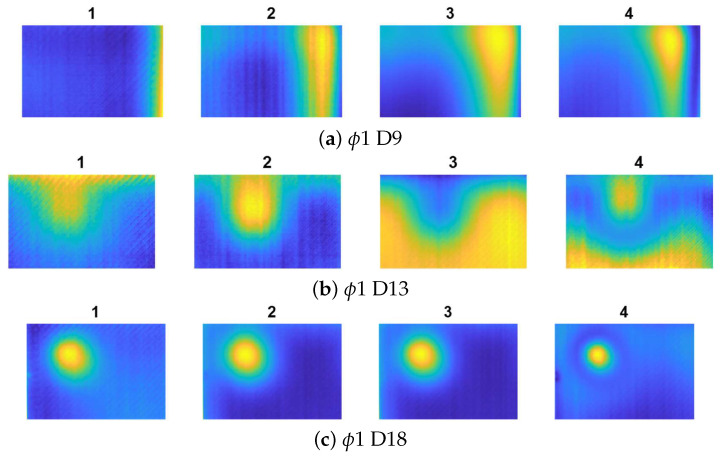
The results of defect visualisation for defects ϕ7 and different D values: 1—raw thermogram (captured after the heating phase t = 60 s), 2—chosen result from sequence $A_{I}$, 3—chosen result from sequence $A_{D}$, and 4—chosen result from sequence $A_{\mathrm{Res}}$.

Figure 7, Figure 8 and Figure 9 show the results for all the cases. Noticeably, for the smallest defects (ϕ1, presented in Figure 7a–c) the defect visualisation algorithm was successful only in the case of deepest flaw (ϕ 1 D18), which is visible in Figure 7c, thereby, showing the best result from the $A_{\mathrm{Res}}$ sequence. For the ϕ4 defects (presented in Figure 8a–c, the localisation of the flaw was impossible only in the case of the shallowest defect (ϕ4 D9—Figure 8a).

For the ϕ7 defects ((presented in Figure 9a–c)), in all cases, the defect visualisation was successful.

**Figure 10:**
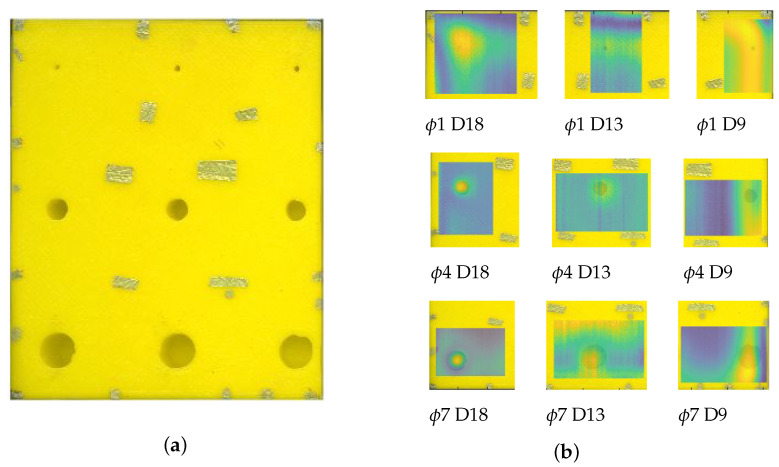
The results of the defect visualisation algorithm presented for the whole sample. (**a**)—the overlaid photos of the front and back of the sample, showing the exact positions of the defects against the position of corresponding markers. (**b**)—chosen best images from the $A_{\mathrm{Res}}$ sequence overlaid on the top of the original photo of the sample.

Figure 10 shows the results for the entire sample. A photo of the back of the sample was overlaid on the top of a photo of its front to indicate the relative location of the defects using markers. The best results from the sequence $A_{\mathrm{Res}}$ were selected for each defect. The resulting signal amplitude distribution was plotted on the original image to show how the result coincided with the actual size and location of the defect (Figure 10b).

## 4. Material State Identification Process

The main idea of the paper was to conduct research enabling the assessment of the possibility of effective extraction of information about even small defects while maintaining short inspection times. Therefore, the aim was to develop an algorithm enabling the recognition of heterogeneity in the examined object based on short-term vector representations of the obtained images. As was described in previous sections, the process of heat transformation in dielectric structures due to their low thermal conductivity is relatively slow.

Therefore, the task of the developed solution based on deep convolutional networks was to search for and recognize even small differences in images. In such an approach, it is not necessary to record the entire complete heating and cooling process and to conduct the assessment, it is enough to acquire several image frames of the material under study.

In this section, we initially describe the preprocessing of the database for the neural network. Thereafter, the system framework is presented in detail, and the concept of a deep neural network and the architecture of the three designed networks are discussed.

### 4.1. Database Preprocessing

The first step of the identification process is database preparation, which contains inputs to feed the deep neural networks. To prepare the set of source data, we used the vectors of 361 original thermal images (acquired during the whole test period) registered for each defect area. By assumption, each frame of the acquired infrared data vector can contain some specific and complex, but partially implicit, information about the defect. Therefore, all the images obtained during the registration of the heating and cooling phases of the tested material were taken for analysis.

**Figure 11:**
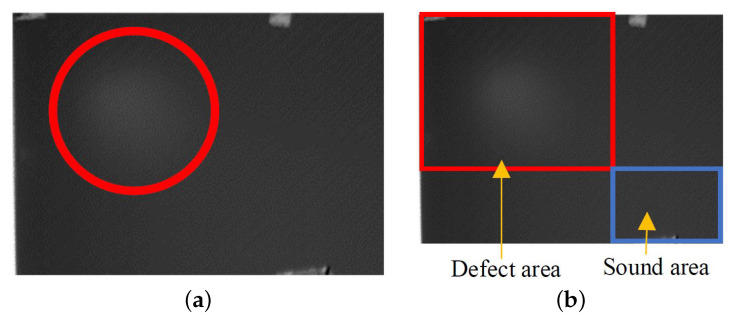
Exemplary results of infrared inspection: (**a**) indication of a defect response, and (**b**) definition of defected and sound areas.

Next, for each defect, its location was indicated. The procedure of defect indication was based on the algorithm of flaw visualisation described in detail in the previous section. As mentioned earlier, in most cases, it was possible to locate the defect with fairly good precision; it was, therefore, possible to extract the defect and sound areas from the original images. Figure 11a shows an exemplary single frame of a thermographic image with the defect location depicted by a red circle.

Upon detecting the flaw location, two areas were cropped from each frame of the infrared images: one representing the defect-free part of the composite, i.e., the defect area marked by a red rectangle and named as class “1”, and one of the sound area, which was marked by a blue rectangle and named as class “0” (as shown in Figure 11b). We did not limit the defect area to the actual defect location, and we selected a sound margin around it. Next, to unify the frame sizes representing both areas in the database, all images were resized (the image size was not considered an effective parameter of the classification).

**Figure 12:**
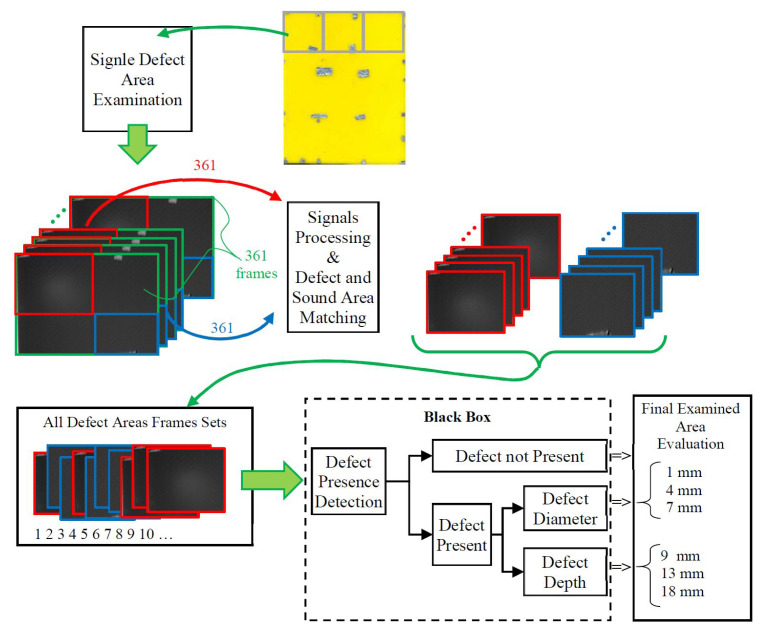
The architecture of the designed system.

Furthermore, the normalisation procedure to the [−1, 1] range was also carried out, where −1 referred to the darkest pixel and 1 to the brightest one. Consequently, for each of the nine artificial defects in the sample, two sub-vectors of 361 frames representing both classes were obtained, which resulted in a total number of 6498 images in the database. A schematic of the procedure is also presented in Figure 12. Additionally, considering the presence of defects of different diameters and depths in the tested sample, two series of subsets were defined within the part of the database related to class 1 (defect presence). The first refers to three possible diameters (1, 4, and 7 mm), while the second indicates three depths (9, 13, and 18 mm) of the observed defects. Consequently, this enabled the achievement of 1083 images per each single case in each subset.

### 4.2. DCNN Based Multi-Label Classification of 3D-Printed Defects

Notably, DCNNs are a branch of classical CNNs with a series of one or more hidden layers that are frequently applied to image processing. Such a deep multi-layered structure is highly efficient and can even generalize very complex problems that they have been trained to solve. The cost of obtaining high application performance is a wide base of possible cases necessary to carry out the proper process of weight optimisation of the ANN. Generally, this consists of three basic components that can be combined into repeating longer sequences, i.e., the convolution, activation function, and subsampling (pooling) layers.

Depending on the target application, there is often an additional fully connected layer and final classification layer as well. The convolutional layers of a DCNN divide an input image into smaller sections and convolve them using a filtering mask to define feature maps, allowing the extraction of critical information. Next, the nonlinear transformation is usually applied by an activation function, such as a hyperbolic tangent or rectified linear unit (ReLu) function, to introduce nonlinearity into the CNN.

Further pooling layers are used, which pool out the most critical information provided by convolutional layers through the implementation of local maximum or average operations. Next, the flattened layer reshapes the data from the two-dimensional feature matrix to the vector, which can be further fed into a fully connected neural network classifier for the final prediction.

In this study, three different architectures were used to enable implementation of the multi-label classification process for the purpose of evaluating the state of the tested material and identifying the occurrence of a defect, its diameter, and depth. Dividing the identification problem into three dedicated structures for a separate task enables a more complete use of the database and maximisation of the accuracy of the classification process. A general diagram of the evaluation process is presented in Figure 12. Evidently, the procedure is designed as a general black-box with cropped images as input and detection/classification results as output. The first step is defect detection: if the image is categorised as non-defect, the process is stopped. Otherwise, it undergoes further processing for diameter and depth classification.

**Figure 13:**
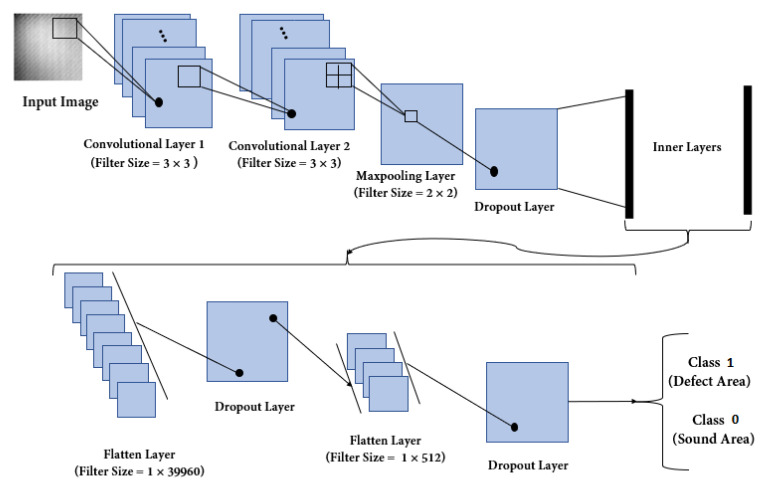
The CNN sequence to classify thermal images.

The general structure of the utilised construction of a DCNN is presented in Figure 13. This structure consists of subsets of convolutional, activation function, pooling layers, and fully connected layers. The structure of the network depends on the number of classes, data size, and features. Considering these characteristics, the structure of the three networks varies partly. The detailed configurations of all three DCNNs are given in Table 2 and Table 3. For the first stage of our system, a deep neural network with two possible classes (defect and non-defect states) was designed.

This network was named the defect detection deep convolutional neural network (3D-CNN), which consisted of two convolution layers with 2D filters of 3 × 3 kernel size, and the maximum operation was used for the pooling layer with a kernel size of 2 × 2. In all the layers, except the last one, the ReLu-type activation function was used. This function stresses the role of nonlinearity, which mostly affects the information content of analysed problems. In the last layer, the sigmoid function was utilised, as this function can be used for binary or multiple classification.

Additionally, the dropout layers were applied only during the training process. This operation prevents neurons in the structure from being conditioned by the output of a particular neuron in the previous layer, by cutting connections between neurons in successive layers (ignoring neurons). Its role is to prevent overfitting of the neural network. The rate parameter given in the tables above refers to the overall scale of connections being deactivated during each iteration, where 1 means 100%.

Furthermore, the classification unit contains two DCNNs: one to establish the detected defect diameter (diameter deep convolutional neural network (DID-CNN)) and one for defect depth estimation (depth deep convolutional neural network (DED-CNN)). The structural difference between 3D-CNN and DID-CNN is based on the filter size of the sigmoid function, as the classification problem refers to three cases. Extracting the depth features is more elaborate than the diameter ones. Hence, we only used two dropout layers in the architecture of the DED-CNN during its training process.

## 5. Results of the Defect Evaluation and Performance Assessment

In this research, we applied codes on Windows 10, and the programming language was Python, Version 3.7. on the Anaconda platform. We used TensorFlow [59] and Keras [60] as our machine learning libraries. Keras is a deep learning API written in Python, running on top of the machine learning platform—TensorFlow. In the designed networks (3D-CNN, DID-CNN, and DED-CNN), 60%, 16%, and 24% of the dataset images were used for training, validation, and testing, respectively. We selected a number of test data larger than the validation data for two main reasons.

First, to have a valid and reliable evaluation of unseen data performance. Second, according to dataset size and features, 16% of the data is adequate for the network to modify its weight to reach an acceptable accuracy, precision, and recall. In Table 4, the total number of images in each category for all networks is shown. In the process of the neural network structure optimization, a stochastic gradient decent, a iterative method with suitable smoothness properties basing on gradient estimation on randomly selected subsets of data, was utilized [61].

In the procedure, a cross entropy was applied as a loss function. One of the challenges in the optimization process is the adjustment of the learning rate, a parameter that is responsible for definition of the response to the estimated error rate during the update of the model’s weights and affecting the overall convergence. The underestimated learning rate can lead to relatively slow convergence rate and, consequently, to a significant increase in the training time.

At the same time, the overestimated learning rate may result in significant fluctuations of the convergence (too wide update steps). Based on preliminary tests, for this study, the learning rate of 0.001 was chosen, which allowed avoiding fluctuations and having logical convergence. Similarly, the number of training epochs was chosen experimentally and set to be equal 50 for all three networks.

### Performance Evaluation

To verify the trained DCNN structures, the classification was run over the testing set. The confusion matrices of all three networks are presented in Table 5 and Table 6. Based on the obtained results, very high levels of correct classification by individual networks were found, which proves the significant potential of the approach. As a result of the obtained results, it is possible to define a number of parameters supporting the assessment process. Furthermore, to evaluate the performance and robustness of the detection and classification networks, the following parameters were considered:

- True Positive (*TP*): expresses all the positive cases that were correctly classified as a positive class in binary classification.
- False Positive (*FP*): expresses all the non-positive cases that were incorrectly classified as a positive class in binary classification.
- True Negative (*TN*): expresses all the negative cases that were correctly classified as a negative class in binary classification.
- False Negative (*FN*): expresses all the non-negative cases that were incorrectly classified as a negative class in binary classification.
- *Accuracy*: refers to the correctly classified cases among all the examined cases; the accuracy is defined as:
  $$Accuracy=\frac{TP+TN}{TP+TN+FP+FN}$$
- *Precision*: refers to the proportion of *TP* to all cases that the network classified as the positive class; the precision is defined as:
  $$Precision=\frac{TP}{TP+FP}$$
- *Recall*: refers to the proportion of *TP* to all positive cases; the recall is defined as:
  $$Recall=\frac{TP}{TP+FN}$$
- Area Under Curve (AUC): measures the area underneath a plot with two parameters: the *TP* rate (as vertical coordinate) and *FP* rate (as horizontal coordinate), and provides an aggregate measure of performance across all possible classification thresholds. Since the *TP* and *FP* rates ranges are between 0 and 1, AUC value is also in the same range and reports the probability that the network correctly classified its input. AUC = 1 (or 100%) means that, with 100% probability, the network will classify the input as the correct class.

In Table 7, the accuracy and the AUC for all proposed networks are shown. Accuracies of 99.42% for the validation set and 99.20% for the test set were reached. This negligible difference between the validation and test shows that the network accurately classified unseen data. In DID-CNN, the accuracies for the validation and test were 99.82% and 98.97%, respectively. For the third network, these numbers were 99.80% and 99.41%.

In the case of the AUC for 3D-CNN, we obtained 99.6% for validation and 99.3% for test data. The AUC values for DID-CNN and DED-CNN were 99.8% and 99.7% for the validation and test data, respectively. The precision and recall of the 3D-CNN are reported in Table 8. The 3D-CNN reached 91.32% for the recall and 96.71% in terms of precision for the validation data, and 91.48% for recall and 96.35% for precision in the test dataset. The results show that all the proposed networks were reliable not only in terms of accuracy but also based on precision and recall.

Concerning the performance evaluation results, an additional test was performed. Additive noise in thermal images is quite prevalent, and one of the most critical challenges of the network is to classify an image when it contains noise. Hence, we evaluated the network performance using images with added noise. The 3D-CNN was trained with a lack of noise images, and then we added noise to the test data and predicted the test data class. Gaussian noise and salt-and-pepper noise were selected as the most practical noise types in thermal images.

**Figure 14:**
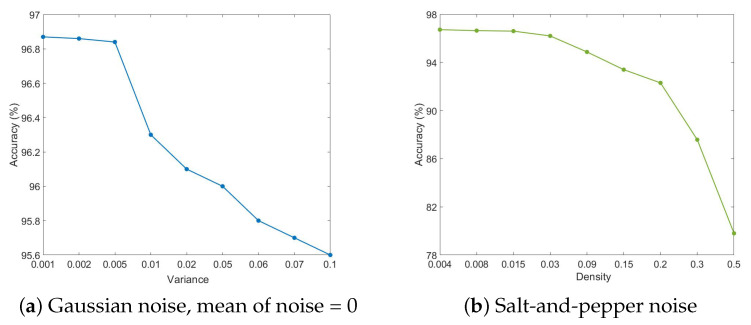
3D-CNN accuracy variation in the noise-added test data.

In Figure 14, we report the accuracy changes while the characteristics of Gaussian and salt-and-pepper noises change. In Figure 14a, the results of the mean accuracy changes can be observed as the variance size is changed. In Figure 14b, the influence of salt-and-pepper noise is reported. The proportion of salt to pepper is 1, and the proportion of noise to pixels is the variable, named density. It can be inferred that the network is persistent against noise. As evident, the 3D-CNN is more vulnerable in the case of salt-and-pepper noise than with Gaussian.

## 6. Conclusions

Defect detection plays a vital role with respect to product control in the 3D printing industry, and is one of the key elements of the final print evaluation. Herein, we proposed a new scheme for defect detection and its chosen parameter classification in ABS 3D printing. The experimental results presented in this study show that active IRT is an adequate tool for evaluating the inner flaws in materials commonly used in AM, and it is envisaged that this method will become one of the techniques commonly used in the non-destructive evaluation of printed materials on an industrial scale.

The main idea of the paper was to conduct research enabling the assessment of the possibility of effective extraction of information about even small defects while maintaining short inspection times. The observation of relatively large areas of the examined object at one time speeds up the inspection but results in a lack of sensitivity to small defects occurring in the examined structure. On the other hand, the use of optical focusing systems (a macro system) allows increasing the local sensitivity but, at the same time, increases the number of required inspections (smaller area of single inspections).

Therefore, the aim was to propose the whole testing procedure enabling both the resolution enhancing macro-lens based data recording and recognition of heterogeneity in the examined object using a short-term vector representations of the obtained images as well. For this purpose, two areas of data processing were presented in this paper. First, a new algorithm of contrast enhancement, based on the curve-fitting and cross-correlation procedure, was presented. The obtained results are promising, and this tool might be useful in the process of analysing experimental data that are characterised by high distortions, which are typically obtained as a result of the thermographic examination of materials with low thermal conductivity.

The proposed signal processing technique is a non-reference method, which increases its versatility and ease of application for various types of experimental data. Contrast enhancement was the first step of the presented study. In the second step, the goal was to evaluate the sample quantitatively, thereby, allowing 3D reconstruction of the examined material. Three separate CNN networks were designed and trained to differentiate between defect and sound areas, as well as to classify the diameters and depths of the detected flaws using only single thermographic image frames of the material under study.

The analysis was based on primary assumptions: the shape and known a priori classes of the defect dimensions. The designed networks were applied to experimental data, and their high accuracy was proven. Moreover, the final performance evaluation procedure, using standard measures, was conducted, indicating over 90% accuracy, precision, and recall for all cases. The last analysis demonstrated the proposed network’s significant immunity to added noise.

The obtained results confirm the possibility of a more effective implementation of the 3D printout state evaluation algorithm on the basis of even short-term inspections (possible for even several frames of thermographic images). The applied deep convolutional network structures confirmed the high potential for detecting even relatively small differences in the registered thermograms. We stress that it is possible to further develop the proposed inspection procedure and extend the scope of the assessment to the identification of different kinds of flaws as well—for example, internal defects.

The thermal signatures of internal defects are significantly different from those obtained for holes. Due to the transmission technique used, the holes were visible on the thermogram as warmer areas, and the internal defects (which are simply an air inclusion in the material) should be visible as cooler areas, as these provide additional insulation for the free flow of heat inside the tested object. Therefore, the difference in thermal signatures will not only allow the detection of the defect using an appropriate neural network but also the classification, i.e., distinguishing the hole from the hidden defect and, thus, the quantitative characterization of the tested object.

Through consideration of the regression problem instead of the classification one in the deep neural network structure, the identification procedure can be extended to defects of any sizes—not only referring to those well defined in the analysed database. Additionally, in order to develop a procedure for an indication of the exact location of defects and to increase the resolution of their localization, we plan to study the possibility of using the time vector representation of temperature distributions at individual points in the studied area as an neural network input. The obtained results will be presented in the future publications.

## Figures and Tables

**Table 1:** Thermal properties of acrylonitrile butadiene styrene (ABS).

Density	Thermal Conductivity	Specific Heat Capacity
(kg/m^3^)	(W/(m·K))	(J/(kg·K))
900–1530	0.15–0.2	1500–1510

**Table 2:** The proposed networks for defect detection (3D-CNN) and diameter classification (DID-CNN).

Operation Layer	Layer Name	Filter Size	Number of Filters	Number of Parameters to Learn
Input	Image Input Layer	Size = 224 × 224 × 3	—	—
Convolutional Layer	Convolution 2D	3 × 3	32	896
Convolutional Layer	Convolution 2D	3 × 3	24	48,408
Pooling Layer	Maxpooling 2D	2 × 2	1	0
Dropout	Dropout	Rate = 0.25	1	0
Inner Layer	Fully Connected Layer	1 × 39,960	1	20,460,032
Dropout	Dropout	Rate = 0.5	1	0
Inner Layer	Fully Connected Layer	1 × 512	1	1026
Dropout	Dropout	Rate = 0.5	1	0
Activation	Sigmoid	2 (3D-CNN)	—	—
		3 (DID-CNN)		

**Table 3:** The proposed networks for depth classification (DED-CNN).

Operation Layer	Layer Name	Filter Size	Number of Filters	Number of Parameters to Learn
Input	Image Input Layer	Size = 224 × 224 × 3	—	—
Convolutional Layer	Convolution 2D	3 × 3	32	896
Convolutional Layer	Convolution 2D	3 × 3	24	48,408
Pooling Layer	Maxpooling 2D	2 × 2	1	0
Dropout	Dropout	Rate = 0.25	1	0
Inner Layer	Fully Connected Layer	1 × 39,960	1	20,460,032
Dropout	Dropout	Rate = 0.5	1	0
Activation	Sigmoid	3	—	—

**Table 4:** The number of training, validation and test images in the presented networks.

Network	Total number	Training	Validation	Test
3D-CNN	361 × 9 × 2 = 6498	3898	1040	1560
DID-CNN and DED-CNN	361 × 9 = 3249	1949	520	780

**Table 5:** Confusion matrix for DID-CNN.

**Validation Set**	**Predicted 1 mm**	**Predicted 4 mm**	**Predicted 7 mm**
Actual 1 mm	165	1	5
Actual 4 mm	0	165	0
Actual 7 mm	0	0	184
**Test Set**	**Predicted 1 mm**	**Predicted 4 mm**	**Predicted 7 mm**
Actual 1 mm	256	4	12
Actual 4 mm	0	255	0
Actual 7 mm	4	0	249

**Table 6:** Confusion matrix for DED-CNN.

**Validation Set**	**Predicted 9 mm**	**Predicted 13 mm**	**Predicted 18 mm**
Actual 9 mm	261	0	2
Actual 13 mm	0	269	0
Actual 18 mm	0	0	248
**Test Set**	**Predicted 9 mm**	**Predicted 13 mm**	**Predicted 18 mm**
Actual 9 mm	373	0	1
Actual 13 mm	0	381	0
Actual 18 mm	0	0	415

**Table 7:** The accuracy and AUC of the three proposed networks.

Network	Validation Accuracy (%)	Test Accuracy (%)	Validation AUC (%)	Test AUC (%)
3D-CNN	99.42	99.20	99.6	99.3
DID-CNN	99.82	98.97	99.8	99.7
DED-CNN	99.80	99.41	99.8	99.7

**Table 8:** The precision and recall of 3D-CNN.

Label	TP	FP	TN	FN	Precision	Recall
Validation	442	15	425	42	96.71%	91.32%
Test	634	24	670	59	96.35%	91.48%

## Data Availability

Not applicable.
